# Tuberous Sclerosis Complex Patients’ Needs and Difficulties—Results of TAND Questionnaire Analysis in Polish Adult Population

**DOI:** 10.3390/jcm11216536

**Published:** 2022-11-03

**Authors:** Anna B. Marcinkowska, Sergiusz Jóźwiak, Agnieszka Tarasewicz, Alicja Dębska-Ślizień, Edyta Szurowska

**Affiliations:** 1 Applied Cognitive Neuroscience Lab, Department of Human Physiology, Medical University of Gdansk, Tuwima Str. 15, 80-210 Gdansk, Poland; 2 2nd Department of Radiology, Medical University of Gdansk, 80-210 Gdansk, Poland; 3 The Children’s Memorial Health Institute, 04-736 Warsaw, Poland; 4 Department of Nephrology, Transplantology and Internal Diseases, Faculty of Medicine, Medical University of Gdansk, 80-210 Gdansk, Poland

**Keywords:** tuberous sclerosis complex, tuberous-sclerosis-complex-associated neuropsychiatric disorders, neuropsychiatric assessment

## Abstract

Introduction: Tuberous Sclerosis Complex (TSC) is a rare genetic disease. Around 90% of individuals with TSC present some neuropsychiatric manifestations (TSC-associated neuropsychiatric disorders, TAND). To date, none of the studies have focused on the TAND profile of the adult population. Thus, the aim of the study was to describe their potential specific needs and difficulties, including differences in cohorts with or without epilepsy and/or intellectual disability. Method: The Polish version of the TAND Checklist was used for assessment of individuals with TSC. Participants had to meet the criteria for diagnosis of TSC. One hundred adult participants (forty-eight males/ fifty-two females; mean age 32.33 ± 11.29) were enrolled in the study. Epilepsy was present in 71% of patients; intellectual disability occurred in a total of 37%. Results: Only 11% of individuals received complete TAND features examination in the past. Moreover, 91.5 of the subjects had four and more TAND symptoms. Intellectually disabled patients and those with epilepsy had more neuropsychiatric problems than epilepsy-free subjects. Conclusions: Findings reveal that TANDs are common in adults with TSC and are underdiagnosed. Most individuals present several behavioural and cognitive problems. Among psychiatric disorders, the most common are ASD, depression, and anxiety disorder. TAND screening should be widely disseminated and applied in clinical practice for early identification, prevention, and rehabilitation of their difficulties. TAND is one of the most significant issues affecting the quality of life of TSC patients and their carers.

## 1. Introduction

Tuberous Sclerosis Complex (TSC) is a rare genetic disease; with an incidence of approximately 1 per 6000 live births [1], it is the second most common neurocutaneous disorder. TSC affects multiple organs (e.g., kidneys, heart, eyes, lungs, brain, and skin) [2]. Clinical features vary widely between patients, making the diagnosis difficult, as this is mostly based on clinical symptoms. New criteria allow for genetic-testing-based diagnosis, even if no other clinical manifestations are observed [3]. Due to the complex nature of TSC, a multidisciplinary approach to diagnosis and treatment is needed at different stages of patient development and life [4,5]. As TSC is a chronic disease, lasting for many years and affecting the whole family, a patient- and family-focused multidisciplinary therapeutic plan should be established [6,7].

To date, there have only been a few studies assessing all varieties of neuropsychiatric manifestations in TSC individuals. Moreover, none of them have focused specifically on the adult population [8,9,10,11,12]. Thus, we focused, in our study, on the psychiatric, psychosocial, emotional, and behavioural problems and specific needs of adults with TSC.

### Neuropsychiatric Manifestations of Tuberous Sclerosis Complex

TSC-associated neuropsychiatric disorder (TAND) is the term combining all neuropsychiatric manifestations of TSC (behavioural, psychiatric, intellectual, academic, neuropsychological, and psychosocial). As each level occurs independently, these abnormalities should be assessed accordingly [13].

Various research works have proved that approximately 40–60% of TSC patients are affected by intellectual disability (IQ < 70) [14,15,16]. Moreover, TSC is related to a high risk of developing neurodevelopmental disorders such as autism spectrum disorder (ASD) (40–50%) [17,18] and attention deficit hyperactivity disorder (ADHD) (30–50%) [5,19]. Forty-eight to fifty percent of individuals with TSC present behavioural issues. Outbursts of anger, severe and prolonged tantrums, hyperactivity, anxiety, impulsiveness, poor focus, self-harm, anxiety and decreased mood are the most commonly seen behavioural disorders in TSC [20]. Moreover, some individuals with TSC develop psychiatric disorders such as depression and anxiety disorders [20]. Data on TSC patients reveal that school issues affect about half of school-age patients with a normal level of intellectual development [20,21]. Mathematics, reading, writing, and spelling issues are the most prevalent [22]. Problems with shifting and maintaining attention, memory recall, spatial memory, and executive functioning are among the cognitive deficiencies in TSC. Self-esteem and self-effectiveness, parental stress and relationship problems are some examples of psychosocial difficulties reported in TSC patients [23].

Despite the fact that TAND symptoms are the most common, they are still rarely diagnosed and treated. The survey conducted among members of the UK Tuberous Sclerosis Association in 2010 showed that only 18% of all included patients received any of the evaluations recommended in the 2005 guidelines [24]. Given that more than 90% of TSC patients will experience neuropsychiatric difficulties at some point in their lives, the gap between clinical need and given interventions is greater than 70% [25]. Thus, the TAND Checklist was developed as a tool to assess all possible concerns facing individuals with TSC. Current guidelines [26,27] recommend regular assessments of cognition and behavioural issues in patients with TSC. The TAND Checklist is a clinical tool for identifying neuropsychiatric features that need further investigation or therapy [20,28]. The TSC Neuropsychiatry Panel created the questionnaire to give a basic framework for a discourse between a clinician and a family or an individual with TSC about TAND [25,29].

Taking into consideration all aspects of TAND symptoms and existing gaps in acknowledgments and treatment, it is necessary to introduce its assessment in everyday practice. Each patient’s TAND profile may change over time, so re-evaluation should be performed regularly. As there are no studies focused specifically on the adult population with TSC, the study aimed to describe their potential specific needs and difficulties, including differences in cohorts with or without epilepsy and/or intellectual disability.

## 2. Materials and Methods

The study protocol was approved by the Ethics Committee of the Medical University of Gdansk (NKEBN/682/2018-2019). All participants and/or caregivers were informed about the study’s merits and signed a written consent form.

### 2.1. Participants

Participants had to meet the criteria for a diagnosis of TSC based on the international diagnostic guidelines [3,30]. Participants needed to be able to complete the TAND Checklist with the researcher or with the help of a parent or caregiver.

### 2.2. Procedure

The Polish version of the TAND Checklist, created by Sergiusz Józwiak and Agnieszka Maryniak, was administered to individuals with TSC and/or their parents or caregivers by a psychologist. The Polish version has been validated and accepted by the authors of the original version. The Polish version contains all of the questions that are contained in the original version [31].

A structured conversation with a parent/caregiver (*n* = 37) or, if possible, directly with the patient (*n* = 63), was conducted “face-to-face”, at a nephrology clinic, during the first-time visit, by a specialist (psychologist). If a patient had an intellectual disability, the interview was conducted with parent/caregiver in the presence of patient, which allowed for a more objective assessment of the patient’s current functional level.

The layout of the questionnaire allows the examiner to detect the existing problem by marking items in a logical order and they may delve further using additional professional diagnostics. The questionnaire is divided into 12 components that follow the TAND levels indicated above: (1) basic developmental milestones; (2) current level of functioning; (3) behavioural concerns; (4) psychiatric disorders diagnosed; (5) intellectual ability; (6) academic skills; (7) neuropsychological skills; (8) psychosocial functioning; (9) parent, caregiver, or self-rating of the impact of TAND; (10) prioritization list; (11) additional concerns; and (12) healthcare professional rating of the impact of TAND.

Most of the questions required simple “Yes” or “No” responses. As the questionnaire lacks a rating scale to determine the intensity of a particular characteristic, when the answer yes was given, more detailed questions were asked to assess needs for a better understanding of the problem, or additional diagnostic or further treatment needs.

The TAND Checklist’s overall structure is shown in Table 1. TAND Checklist is publicly available for free in several language versions online at: https://tandconsortium.org/checklists/ (accessed on 30 August 2022).

In our study, the overall time of the interview varied between 25 and 45 min, depending on how many additional issues and needs were observed.

### 2.3. Statistical Analysis

Statistical Package for the Social Sciences (SPSS AMOS 27 IBM, Corp., Armonk, NY, USA) for Windows was used for data analysis. Means ± standard deviation (SD) for quantitative variables and absolute counts and frequencies for qualitative variables were used. The normality of the distributions of the quantitative variables was verified by applying the Shapiro–Wilk test. The Chi-square test was used for categorical variables. The Student t-test was used for normally distributed groups, and the Mann–Whitney U-test was applied for not normally distributed variables. We considered a two-tailed *p* value of 0.05 or less to be statistically significant. For assessing multiple dependent variables simultaneously multivariate analysis (MANOVA) was performed.

## 3. Results

A total of 100 adult participants (48 males/52 females) were enrolled in the study. The mean age of the participants was 32.33 ± 11.29. Epilepsy was present in 71% of patients and 31% had experienced infantile spams in the past. Intellectual disability (borderline to profound) occurred in a total of 44% of subjects, of which 16.7% had borderline (IQ 70–80), 21.4% mild (IQ 50–69), 26.2% moderate (IQ 35–49), 14.3% severe (IQ 21–34) and 26.2% profound (IQ < 20) intellectual disability.

The characteristics of the study group are presented in Table 2.

In general, the most common symptoms on a behavioural level were temper tantrums (61%), difficulties in paying attention (59%) and getting on with people of similar ages (58%), impulsivity (57%), and anxiety (54%). The most common psychiatric disorders were ASD (22%), anxiety (19%), and depressive (13%) disorders and the most problematic scholastic skill was mathematics (52%). On a neuropsychological level, four out of six cognitive functions were problematic in more than half of individuals with TSC. On a psychosocial level, the most common symptom was low self-esteem (52%). All of the detailed results are presented in Table 3 and Table 4.

There was a statistically significant difference in the coexistence of intellectual disability, autism, and epilepsy based on patients’ IQ F(hypothesis df, Error df) = F value, *p* < sig; Wilk’s Λ = value, partial η2 = partial eta squared (F(2,97) = 25.052, *p* < 0.001; Wilk’s Λ = 0.659, partial η2 = 0.341).For a better understanding of the various cohorts of TSC individuals studied, the results were also analysed despite the presence/absence of epilepsy and normal IQ/intellectual disability.

Comparing epilepsy to the epilepsy-free groups, statistically significant differences were observed in the frequency of depressed mood (*p* = 0.03), speech delay (*p* = 0.002), difficulties getting on with other people of similar ages (*p* = 0.001), paying attention (*p* = 0.042) and impulsivity (*p* = 0.001). ASD was only observed in the epilepsy group, although anxiety disorder was more often diagnosed in epilepsy-free individuals than in individuals with epilepsy. Patients with epilepsy more often had difficulties than the epilepsy-free group within all neuropsychological functions. Although, statistically significant differences between the mentioned groups were observed in executive functioning (*p* = 0.035) and getting disoriented (*p* = 0.001). Family stress (*p* = 0.005) and relationship problems were more often reported by individuals without epilepsy than by epileptic subjects (*p* = 0.005). The comparison of TAND results in the epilepsy and epilepsy-free groups is presented in Table 3.

In the comparison of intellectually disabled/normal IQ groups, statistically significant differences were observed in anxiety (*p* = 0.045) and depressed mood (*p* = 0.045), self-injury (*p* = 0.05), difficulties getting on with other people of similar ages (*p* = 0.001) and paying attention (*p* = 0.045), restlessness or fidgetiness (*p* = 0.005) and impulsivity (*p* = 0.05). In the intellectually disabled group, almost half of the subjects were diagnosed with ASD, and did not develop mathematical or spelling skills. On a neuropsychological level, the biggest difference was observed in attention (*p* = 0.037), executive functions (*p* = 0.05) and getting disoriented (*p* = 0.032). Family stress (*p* = 0.035) and relationship difficulties (*p* = 0.002) were more frequently observed in individuals with normal IQ than in intellectually disabled group. The comparison of TAND results in the normal IQ and intellectually disabled groups is presented in Table 4.

In the ninth question of TAND, individuals were asked to rate how much the difficulties stated bother them on a scale of 1 to 10. The mean caregiver in the general group was 5.87 (intellectually disabled—6.6, normal IQ—5.3, epilepsy—6, and epilepsy-free—5.1).

The twelfth question was completed by the person who conducted the TAND checklist examination (psychologist) and concerned the impact of TAND on the individual and their family (on a scale from 1 to 10). The mean specialist rate for the general group was 5.1 (intellectually disabled—6, normal IQ—4.4, epilepsy—5.3, and epilepsy-free—4.5).

Statistically significant differences (calculated with the Mann–Whitney U-test) between subjective (patient/caregiver) and objective (specialist/psychologist) rates were observed in the normal IQ (difference = 0.9) and epilepsy (difference = 0.75) groups. Detailed results are presented in Table 5.

According to the TAND interview, only 11% of individuals had ever completed the TAND Checklist and those who had were the youngest subgroup of a studied cohort (18–21 years old). Only 36% had ever received psychological or psychiatric evaluation and almost none had ever had a detailed neuropsychological assessment. Scholastic difficulties were the most often examined symptoms assessed in 56% of subjects. The TAND Checklist revealed that 89% of the examined subjects had at least four TAND features (more in the intellectually disabled group—95.2%). Most symptoms were reported in patients in the intellectual disability (m = 14.5) and epilepsy groups (m = 14.8). Detailed results are presented in Table 6 and Table 7.

The more frequent TAND problems detected in our cohort were (at the behavioural level): difficulty paying attention (*n* = 20), extreme shyness (*n* = 29), mood swings (*n* = 21), repeating words or phrases over and over again (*n* = 22), difficulty getting on with peers (*n* = 20), being very rigid or inflexible about how to do things (*n* = 26), and restlessness or fidgetiness (*n* = 22). At the psychiatric level, anxiety disorders (*n* = 10) prevailed. At an intellectual level, intellectual disability affected 15 cases, while difficulties with writing and mathematics prevailed at the academic level. At the neuropsychological level, 14 patients showed difficulties in multitasking and 10 in cognitive flexibility. Finally, at the psychosocial level, 14 cases had difficulties in social interaction.

Several additional problems, not included in TAND Checklist, were mentioned by the interviewed subjects.

In an in-depth interview, several subjects from the normal-IQ group mentioned a fear of having children, as well as feelings of guilt related to the transmission of the TSC gene to their descendants. This was especially related to individuals with newly confirmed TSC (*n* = 8), some of whom were diagnosed after their child received a diagnosis. Half of the interviewed patients within the normal IQ (*n* = 28) range reported relationship problems such as being separated, divorced or having intimate relationships with other people related to low self-esteem and feelings of shame. One of the additional issues mentioned by subjects in our study was relationship difficulties, related to sexual problems, which were mostly associated with a fear of getting pregnant and the feelings of shame and low self-esteem caused by skin lesions. Moreover, psychologists noted low levels of sexual education, especially within the intellectually disabled group. Adding sexual education specialists, especially for the young adult group, might help with some of their needs.

In additional issues, patients within the normal IQ range mentioned occupational problems related to concentration difficulties (*n* = 14), as well as getting hired because of their epilepsy (*n* = 7).

ASD symptoms were noticed in eight individuals with normal IQ and without psychiatric diagnosis.

Additional psychiatric assessment was referred in eight cases. Three were diagnosed with depressive disorder, three with anxiety disorder, one with bipolar disorder and one with dissociative disorders (dissociative neurological symptom disorder, with non-epileptic seizures and dissociative amnesia).

Furthermore, 23% of subjects with normally distributed IQ reported suicidal thoughts and 11% attempted suicide (some of them multiple times).

## 4. Discussion

TSC has become the subject of more intensive research in the last decade of the twentieth century. Despite this, knowledge of neuropsychiatric illnesses remains insufficient to describe a patient’s functioning profile. Similar to the wide variety of physical symptoms, neuropsychiatric symptoms of TSC differ between patients; thus, each individual with TSC has their own unique TAND profile [20,28].

Our research aimed to identify all symptoms that concern adult TSC patients and their families; in the future, this may allow a personalised intervention plan focused on their needs to be created. During the diagnostic procedure, many of these abnormalities are ignored. Previous research highlights the fact that, although TANDs appear in the vast majority of patients, less than 20% of patients with TSC are properly diagnosed and receive the necessary help [25,32]. Our results show that when considering only adult populations, the detailed TAND assessment is even rarer. In our study, only 11% of individuals ever received a complete TAND features examination and those who had were the youngest of our cohort. This emphasises the fact that a greater gap exists in the treatment of TAND symptoms in adult patients. In our study, 89% of subjects mentioned more than four TAND symptoms (the mean count of TAND symptoms was 12.8) and only 36% ever received any form of psychological or psychiatric help.

A recent multidisciplinary study of TSC, the Tuberous Sclerosis registry to increase disease Awareness (TOSCA), showed that 44.4% of participants were within the normal range of intellectual abilities (47% within adults). In our study, 37% of subjects had a diagnosis of intellectual disability. A lower count of intellectually disabled patients in our study than in the TOSCA study might be explained by the source of subjects (nephrology clinics). It is plausible that some adult patients with an intellectual disability did not yet present the necessity for treatment of renal manifestations of TSC. Another possible explanation is that, due to the pandemic (the study was conducted between July 2017 and July 2021), some patients had logistic problems with access to healthcare.

In our study, more than half of individuals had behavioural problems such as temper tantrums, difficulties in paying attention and getting on with other people of similar ages, impulsivity, and high levels of anxiety. The most common psychiatric diagnosis was ASD, in 22% of individuals. The identification and acknowledgment of behavioural issues should lead to the identification of possible causes of behavioural challenges and the execution of suitable therapeutic procedures, although the minority of patients received needed psychiatric and therapeutic help.

Affective disorders such as anxiety and depressive disorders are underdiagnosed among the studied population, as high levels of anxiety were the most common affective symptom and depressive symptoms concerned 1/3 of subjects. In our study, eight patients were referred for further psychiatric diagnosis (three were diagnosed with depressive disorder, three with anxiety disorder, one with bipolar disorder and one with dissociative disorders—dissociative neurological symptom disorder, with non-epileptic seizures, and dissociative amnesia). In all eight cases, psychopharmacotherapy was introduced. Furthermore, psychotherapy was recommended for improving their mental health status.

Although scholastic skills concerned the majority of TSC individuals, among the youngest group, 67% received necessary help, such as individual school plans. Similar to an intellectual development diagnosis, 89% of intellectually disabled subjects received a formal IQ assessment. Even though, subjectively, four out of six cognitive functions were problematic in more than half of the TSC individuals studied, none of them ever received a formal neuropsychological assessment in their adult life. This underlines the greatest gap in the currently existing diagnostic system.

Moreover, TAND symptoms differ between patient cohorts (e.g., with and without intellectual disability and/or epilepsy). Our findings showed that epilepsy-free individuals more often report a depressed mood. Similarly, anxiety disorder was more often diagnosed in the epilepsy-free group. This might be linked to a potential positive impact of antiepileptic drugs on mood [33]. Similar results were observed in the comparison of intellectually disabled and normal-IQ subjects in which individuals with an IQ within the normal range had higher levels of depression than intellectually disabled subjects, possibly linked to the subjective higher rating TAND impact on everyday life. Toldo et al. [9] claimed that those findings support the hypothesis that patients with a normal IQ could have a better awareness of their pathology that favours the development of an internalising and affective disorder. It is worth considering that the diagnosis of depression in intellectually disabled patients is more challenging, especially in those who did not develop or have a speech delay. Nevertheless, it seems that affective problems are underdiagnosed in all adult groups and it should be noted that subjects with TSC have to be diagnosed by psychiatrists with a special focus.

The results of our study showed that patients with epilepsy and those who are intellectually disabled more often had difficulties with all neuropsychological functions than epilepsy-free individuals, especially concerning executive functioning and getting disoriented. It is widely known that epilepsy itself causes both disturbances in cognitive development and neuropsychological deficits, which leads to encephalopathy [34,35,36,37].

Our results showed that intellectually disabled patients with TSC have more behavioural problems, such as self-injury, difficulties getting on with other people of similar ages and paying attention, restlessness or fidgetiness and impulsivity. This is related to the fact that almost half of the subjects in the intellectually disabled group were diagnosed with ASD and those problems are common within the spectrum. However, it is worth noting that 23% of subjects with normally distributed IQ reported suicidal thoughts and 11% attempted suicide (some of them multiple times). Moreover, family stress and relationship difficulties were more frequently observed in normal IQ and epilepsy-free individuals than in intellectually disabled and epileptic groups. To date, there has been no report describing the frequency of suicide issues in TSC. Based on our research, we noticed that suicide is one of the most important psychiatric issues and its addition to the revised version of the TAND Checklist should be considered.

Family stress and relationship problems were more often reported by individuals without epilepsy than with epilepsy. In an in-depth interview, those individuals mentioned a fear of having children more often, as well as feelings of guilt related to the transmission of the TSC gene to their descendants. This was especially related to individuals with newly confirmed TSC, some of whom were diagnosed after their child received a diagnosis. Half of the interviewed patients within the normal IQ range reported relationship problems such as being separated, divorced or having intimate relationships with other people related to impulsivity, low self-esteem, and feelings of shame. In additional issues, patients within the normal IQ range mentioned occupational problems related to concentration difficulties, as well as getting hired because of their epilepsy. Adding another question about work status to TAND Checklist might therefore be useful. Caregivers of intellectually disabled individuals mentioned the incapacitation of their children and worries about the future care of TSC patients related to ageing [38]. All of those aspects show that social care providers should also be introduced to the therapeutic team.

Several additional problems, not included in the TAND Checklist, were mentioned by the interviewed subjects. Some adults with TSC faced different types of problems, such as pregnancy and the diagnosis of TSC in their child. Families who have learned of their child’s illness face a stressful situation and require psychological assistance. Later on, they will need a lot of help, as well as attention for their problems and information about possible sources and forms of assistance. Families who face their children’s disorders without sufficient help end up facing separation and divorce. Adding the age of diagnosis and more detailed questions about family status and TSC in children to the TAND Checklist might help to find potential fields of special needs. Few patients learned about their diagnosis after having a child with more eminent features; all of them faced feeling of guilt and fear.

One of the additional issues mentioned by subjects in our study was relationship difficulties, related to sexual problems, which were mostly associated with a fear of getting pregnant and the feelings of shame and low self-esteem caused by skin lesions. Moreover, psychologists noted low levels of sexual education, especially within the intellectually disabled group. Adding sexual education specialists, especially for the young adult group, might help with some of their needs.

The minority of individuals with TSC may never have any TAND problems. However, it is important to consider that TAND symptoms may arise later in life despite years of apparently “normal” functioning. The diversity of psychopathologies that influence each other makes the accurate diagnosis of neuropsychiatric symptoms extremely difficult. All risk areas must be properly diagnosed and patients must receive the required psychological and psychiatric care, as well as effective treatments for their individual illnesses. Our findings based on the TAND Checklist showed that neuropsychiatric features in adult patients with TSC have to be properly assessed. Detailed, structural, deepened interviews with patients and/or their caregivers are needed for the best practice allowing for precise additional diagnostic testing or specialised counselling; describing the mechanism of challenges or providing information on therapy centres that deal with a specific problem are both examples of person-centred interventions.

## 5. Conclusions

To the best of our knowledge, this is the first such study focused specifically on an adult population with TSC. The study describes adult TSC patients’ specific needs and difficulties, including differences in cohorts with or without epilepsy and/or intellectual disability. The following major problems were identified:

(1) TANDs are common in TSC and are underdiagnosed. Almost 90% of patients present more than four TAND symptoms and less than 40% ever received any form of psychological or psychiatric help.
(2) The most common behavioural symptoms are temper tantrums, difficulties in paying attention, and getting on with people of similar ages, impulsivity, and anxiety. The most common psychiatric disorders are ASD, depression, and anxiety disorder.
(3) The majority of patients develop cognitive problems in multiple domains.
(4) A detailed diagnosis of scholastic and cognitive problems is needed to introduce specific help matched for TSC patients’ needs.
(5) The epilepsy and intellectually disabled group presents more neuropsychiatric symptoms.
(6) One of the crucial needs of the adult TSC patients is regular psychiatric assessment to prevent affective disorders as well as suicidal attempts.
(7) TSC in the adult population is associated with parental stress, intimacy and relationship issues as well as occupancy difficulties. TAND is one of the most significant issues affecting the quality of life of TSC patients and their carers.

As the TAND is one of the most common TSC features, neuropsychiatric screening should be widely disseminated and utilised in clinical practice for early identification, prevention and rehabilitation. Each patient’s TAND profile may change over time, so re-evaluation should be performed on a regular basis.

## Figures and Tables

**Table 1:** TAND Checklist—structure.

TAND—Checklist	Neuropsychiatric Domain
**1.(A–G)**	Development—milestones
**2.(A–C)**	Current functioning level
**3.(A–S)**	Behavioural and affective symptoms
**4.(A–F)**	Diagnosed psychiatric and neurodevelopmental disorders
**5.(A–D)**	Intellectual abilities
**6.(A–D)**	School difficulties
**7.(A–F)**	Cognitive functioning
**8.(A–C)**	Psychosocial functioning
**9.**	Subjective rating of the impact of TAND symptoms on life
**10.**	Priority issues
**11.**	Additional issues
**12.**	Rating of the severity of TAND symptoms according to the examiner

**Table 2:** Clinical characteristics of the TSC Cohort.

TSC Patients	(n = 100)	
Mean age (years) ± SD	32.33 ± 11.29	
Median age	32	
Gender	Male 48	Female 53
Diagnostic criteria met for TSC	100 (100%)	
Cortical malformations	96%	
Subependymal nodules (SEN)	70%	
Subependymal giant cell astrocytoma (SEGA)	17%	
Never had epilepsy	29%	
Infantile Spasms	31%	
Normal IQ	56%	
Borderline IQ	7%	
Mild ID	9%	
Moderate ID	11%	
Severe ID	6%	
Profound ID	11%	
Autism Spectrum Disorder	22%	

IQ—Intelligence Quotient, ID—Intellectual Disability.

**Table 3:** TAND results in the studied group (*n* = 100) and with division in view of epilepsy.

TAND LEVEL	TAND Feature		Frequency (%)		Χ^2^	*p*
		All *n* = 100	Epilepsy *n* = 71	Epilepsy Free *n* = 29		
Behavioural	Anxiety	54	57.6	46.4	0.213	0.718
	Depressed mood	34	**26.3**	**53.7**	**6.565**	**0.032**
	Extreme shyness	24	23.9	20.7	0.012	0.913
	Mood swings	64	59.2	68.9	2.498	0.086
	Aggressive outbursts	38	52.1	51.7	0.718	0.275
	Temper tantrum	61	57.7	58.6	0.312	0.592
	Self-injury	34	33.8	34.5	1.483	0.309
	Absent or delayed speech	51	**54.9**	**37.9**	**5.689**	**0.002**
	Repeating words or phrases over and over again	18	15.5	24.1	2.730	0.249
	Poor eye contact	39	39.4	34.5	1.375	0.451
	Difficulties getting on with other people of similar age	58	**63.4**	**37.9**	**9.308**	**0.001**
	Repetitive behaviours	44	46.5	37.9	3.714	0.089
	Very rigid or inflexible about how to do things	40	47.8	34.5	2.381	0.165
	Overactivity/hyperactivity	35	36.6	31	3.034	0.097
	Difficulties paying attention	59	**64.8**	**34.5**	**7.934**	**0.042**
	Restlessness or fidgetiness	49	53.5	37.9	1.974	0.503
	Impulsivity	57	**63.3**	**37.9**	**12.047**	**0.001**
	Difficulties with eating	37	38	51.7	4.047	0.076
	Sleep difficulties	50	50.1	49.4	0.936	0.760
Psychiatric	ASD	22	29.6	0	4.054	0.068
	ADHD	2	2.8	0	1.043	0.247
	Anxiety disorder	19	18.3	20.7	0.083	0.878
	Depressive disorder	13	15.5	10.3	1.002	0.309
	Obsessive Compulsive Disorder	3	2.8	3.4	3.043	0.094
	Psychotic disorder	2	2.8	0	1.032	0.610
Intellectual	Intellectual disability (borderline (IQ 70–80); mild (IQ 50–69); moderate (IQ 35–49); severe (IQ 21–34); profound (IQ < 20))	41 (7;9;11;6;11)	**53.4 (7;9;11;6;11)**	**6.9 (2;0;0;0;0)**	**11.943**	**0.003**
Academic	Reading	11 (16 n/a)	18.3 (14.1 n/a)	17.2	1.073	0.603
	Writing	7 (20 n/a)	14.1 (18.3 n/a)	13.8	2.043	0.395
	Spelling	20 (31 n/a)	32.4 (22.5 n/a)	34.5	1.043	0.495
	Mathematics	52 (28 n/a)	39.4 (36.6 n/a)	55.2	3.485	0.273
Neuropsychological	Memory	61	63.3	48.3	3.065	0.089
	Attention	57	62	37.9	5.054	0.075
	Dual tasking	58	57.7	48.3	3.065	0.089
	Visuo-spatial tasks	29	43.7	27.6	5.054	0.075
	Executive skills	57	**70.4**	**37.9**	**8.043**	**0.035**
	Getting disoriented	27	**49.3**	**8.4**	**13.054**	**0.001**
Psychosocial	Low self-esteem of the patient	52	49.3	48.3	1.065	0.289
	Family stress	41	**38**	**51.7**	**6.256**	0.005
	Relational difficulties of the patient	41	**38**	**48.3**	**6.058**	0.005

ASD—Autism Spectrum Disorder; ADHD—Attention Deficit Hyperactivity Disorder; IQ—Intelligence Quotient; n/a—not applicable (subject did not develop skill yet).

**Table 4:** TAND results in the studied group (*n* = 100) and with division in view of intellectual disability.

TAND LEVEL	TAND Feature	Frequency (%)			Χ^2^	*p*
		All *n* = 100	Intellectually Disabled *n* = 44	Normal IQ *n* = 56		
Behavioral	Anxiety	54	**67**	**44.8**	**7.585**	**0.045**
	Depressed mood	34	**21**	**43.1**	**7.375**	**0.045**
	Extreme shyness	24	28.6	20.1	1.367	0.668
	Mood swings	64	64.3	63.8	0.422	0.789
	Aggressive outbursts	38	54.8	50	1.479	0.675
	Temper tantrum	61	69	55.2	3.276	0.434
	Self-injury	34	**42.9**	**27.6**	**6.934**	**0.05**
	Absent or delayed onset of language to communicate	51	61.9	43.1	2.658	0.564
	Repeating words or phrases over and over again	18	14.3	20.7	2.832	0.467
	Poor eye contact	39	40.5	37.9	1.732	0.589
	Difficulties getting on with other people of similar age	58	**73.8**	**46.5**	**11.04**	**0.001**
	Repetitive behaviours	44	42.9	44.8	0.327	0.967
	Very rigid or inflexible about how to do things	40	47.6	41.4	1.264	0.756
	Overactivity/hyperactivity	35	33.3	36.2	0.092	0.879
	Difficulties paying attention	59	**73.8**	**48.3**	**7.496**	**0.045**
	Restlessness or fidgetiness	49	**64.3**	**37.9**	**8.579**	**0.005**
	Impulsivity	57	**71.4**	**46.6**	**6.249**	**0.05**
	Difficulties with eating	37	35.7	37.9	0.383	0.879
	Sleep difficulties	50	52.4	48.3	0.335	0.878
Psychiatric	ASD	22	**47.6**	**3.4**	**13.397**	**0.001**
	ADHD	2	2.4	1.7	0.285	0.898
	Anxiety disorder	19	21.4	17.2	0.184	0.987
	Depressive disorder	13	16.7	10.3	0.215	0.876
	Obsessive Compulsive Disorder	3	0	5.2	1.267	0.657
	Psychotic disorder	2	4.8	0	0.157	0.889
Intellectual	Intellectual disability (borderline (IQ 70–80); mild (IQ 50–69); moderate (IQ 35–49); severe (IQ 21–34); profound (IQ < 20))	41 (7;9;11;6;11)	**100 (7;9;11;6;11)**	**0**	**13.948**	**0.001**
Academic	Reading	11 (16 n/a)	23.8 (26.2 n/a)	10.3	1.158	0.658
	Writing	7 (20 n/a)	30.9 (16.7 n/a)	12.1	3.268	0.345
	Spelling	20 (31 n/a)	30.9 (47.6 n/a)	21	2.572	0.567
	Mathematics	52 (28 n/a)	40.5 (47.6 n/a)	60	2.256	0.556
Neuropsychological	Memory	61	66.7	56.9	1.257	0.739
	Attention	57	**78.6**	**41.4**	**7.634**	**0.037**
	Dual tasking	58	64.3	53.4	2.043	0.276
	Visuo-spatial tasks	29	45.2	34.5	2.143	0.246
	Executive skills	57	**78.6**	**53.4**	**6.952**	**0.05**
	Getting disoriented	27	**54.8**	**21.4**	**7.843**	**0.032**
Psychosocial	Low self-esteem of the patient	52	52.4	51.2	0.002	0.846
	Family stress	41	**28.8**	**50**	**7.732**	**0.035**
	Relational difficulties of the patient	41	**23.8**	**53.4**	**9.522**	**0.002**

ASD—Autism Spectrum Disorder; ADHD—Attention Deficit Hyperactivity Disorder; IQ—Intelligence Quotient; n/a—not applicable (subject did not develop skill yet).

**Table 5:** Mean patient and specialist rate of TAND symptoms’ impact.

	Mean Patient/Caregiver Rate (sd)	Mean Psychologist Rate (SD)	Mean Difference
*all*	5.87 (2.69)	5.1 (2.08)	0.77
*Intellectually disabled*	6.6 (2.17)	6 (2.26)	0.57
*normal IQ*	5.3 (2.9)	4.4 (1.64)	**0.9** **
*epilepsy*	6 (2.63)	5.3 (2.21)	**0.75** *
*epilepsy free*	5.1 (2.81)	4.5 (1.53)	0.62

IQ—Intelligence Quotient; * *p* < 0.05, ** *p* < 0.001.

**Table 6:** Frequency of patients who reported 4 and more TAND symptoms in all groups.

	*Frequency (%) of 4 and More TAND Symptoms*		
	Intellectual Disability	Normal IQ	All
*Epilepsy*	95	87.1	91.5
*No epi*	100	77.8	79.3
*All*	95.2	84.5	89

**Table 7:** Mean and median count of TAND symptoms reported in all groups.

	Mean Count of TAND Symptoms (SD)	Median Count of TAND Symptoms
*All*	12.8 (6.76)	12
*Intellectually disabled*	14.8 (6.26)	15.5
*Normal IQ*	10.1 (5.97)	8
*Epilepsy*	14.5 (7.24)	14
*No epi*	10.6 (7.44)	9

IQ—Intelligence Quotient.

## Data Availability

Data will be available on demand.
